# Investigation of salt stress effects on maize seedling phenotypic traits based on the PointCornNet point cloud segmentation model

**DOI:** 10.3389/fpls.2025.1621509

**Published:** 2025-09-09

**Authors:** Xiaozhong Li, Zhiqian Ouyang, Qianzhe Cheng, Zhibo Zhong, Xiuqing Fu

**Affiliations:** ^1^ College of Mechanical Engineering, Yangzhou Polytechnic College, Yangzhou, China; ^2^ College of Engineering, Nanjing Agricultural University, Nanjing, China; ^3^ Institute of Farmland Water Conservancy and Soil-Fertilizer, Xinjiang Academy of Agricultural and Reclamation Science, Shihezi, Xinjiang, China

**Keywords:** maize seedlings, PointNet model, phenotypic detection, salt stress, point cloud segmentation

## Abstract

To address the limitations of traditional crop phenotyping methods, such as slow data collection, high error susceptibility, and seedling damage, we proposed a non-destructive approach for phenotypic trait detection in maize seedlings to enhance breeding efficiency. We developed an improved point cloud segmentation model, PointCornNet, based on PointNet++, by integrating the CBAM attention mechanism, replacing the original loss function with Varifocal Loss, and incorporating the CronDBSCAN clustering algorithm to enhance segmentation accuracy and enable both semantic and instance segmentation. Comparative experiments confirmed the improved model performance. Phenotypic parameters—including plant height, canopy width, volume, and surface area—were calculated from the segmented point clouds. The coefficient of determination (R²) between calculated and manually measured values for plant height and canopy width reached 0.99 and 0.96, respectively, demonstrating the accuracy of the method and non-destructive nature. Using PointCornNet and the phenotyping algorithm, we measured 3D morphological changes of maize seedlings under different NaCl concentrations during the first six days after sowing. The results showed that salt stress significantly inhibited seedling growth, with stronger inhibition at higher NaCl concentrations. Increased salt concentration delayed initial seedling emergence and led to gradual decreases in plant height, canopy width, volume, surface area, and their respective growth rates.

## 1 Introduction

Plant phenotypes are determined by the combined influence of genotype and environment, and refer to the observable characteristics of plants under specific environmental conditions, including morphology, structure, physiological traits, and growth status (Cooper and DeLacy, 1994). Measuring specific and well-defined physiological phenotypes is of great importance for understanding growth processes, estimating yield, evaluating disease resistance, and advancing breeding programs (Li et al., 2021). Phenotypic measurement of seedlings is particularly critical for both breeding and seed testing. During the breeding process, growth traits of seedlings aid in the early selection of superior individuals, thereby enhancing breeding efficiency. Analysis of morphological features and leaf structure provides a basis for variety improvement. At the same time, seedling phenotyping can assess seed quality and ensure germination rates and growth performance under different environmental conditions, ultimately improving seed stability and adaptability. Plant height is one of the key indicators of plant growth and development; accurate and rapid estimation of plant height can facilitate and improve crop yield prediction (Varela et al., 2017). Canopy width is an important parameter for assessing plant growth status and photosynthetic potential (Guo et al., 2005). Crop volume reflects the plant’s spatial occupancy, growth condition, and biomass accumulation (Xiao et al., 2020). The surface area of crops directly determines their ability to capture light energy; a larger leaf area increases the photosynthetic surface, promotes dry matter accumulation, and provides more energy for seedling development (Yang et al., 2009).

Maize is the third most important cereal crop after rice and wheat (Farooq et al., 2015), primarily composed of starch, protein, and fat, and is also rich in micronutrients such as vitamin A, vitamin E, and selenium (Jiao et al., 2022). It can be processed into a variety of industrial products including starch, sweeteners, oil, beverages, adhesives, industrial alcohol, and bioethanol (Ranum et al., 2014), and it also plays a vital role in the livestock and poultry industries (Loy and Lundy, 2019). With increasing environmental degradation and climate change, the impact of abiotic stress on plant growth and productivity has intensified (Chieb and Gachomo, 2023), among which salt stress is one of the most significant abiotic factors disrupting plant development. Several studies have investigated the effects of salt stress on maize growth. For example (Sun et al., 2022), examined its influence on germination rate, radicle and coleoptile length, and the dry matter content of radicles and coleoptiles; (Turan et al., 2009) reported that NaCl reduced the dry biomass of maize plants, increased stomatal resistance and proline concentration, and decreased chlorophyll content. However, there is limited research on the effects of salt stress on the phenotypic traits of maize seedlings. Investigating these effects could provide experimental support for breeding salt-tolerant varieties and lay the groundwork for sustainable agricultural development.

At present, plant phenotyping has become a research hotspot in agriculture, as studying plant phenotypes under different environmental conditions contributes to genetic screening and field yield assessment of crops (Li et al., 2021). Traditional phenotypic measurements are primarily conducted manually, which often suffer from low efficiency, destructiveness, and subjective errors. These conventional methods are also constrained by the limitations of the tools used and the surrounding environmental conditions, making large-scale, high-precision measurements challenging. Therefore, in modern breeding and cultivation trials, there is an urgent need to develop advanced techniques for phenotypic data acquisition.

In recent years, two-dimensional image-based phenotyping has been widely applied (Gehan et al., 2017) (Hu et al., 2018). conducted high-throughput phenotypic analysis of sorghum plant height by capturing RGB images of sorghum fields using unmanned aerial vehicles (UAVs) and applying a self-calibrating method (Weizheng et al., 2020). constructed a plant skeleton model using a skeleton-thinning algorithm, detected endpoints and branching points on the skeleton, and analyzed the structure through a binary tree approach, ultimately identifying hierarchical nodes and individual leaves to achieve segmentation of maize stems and leaves (Bylesjö et al., 2008). developed LAMINA, a tool for automatic analysis of leaf image features such as leaf size, area, asymmetry, serration, and missing regions. The tool’s accuracy was validated using a dataset of European aspen leaf images.

For more complex plant structures or when measuring multiple phenotypic traits of a given species simultaneously, three-dimensional reconstruction offers greater advantages (Guan et al., 2018). developed a low-cost and efficient imaging system using an RGB camera and a photonic mixer device (PMD) sensor to reconstruct 3D models of soybean plants, thereby obtaining canopy information (Sun et al., 2017). measured cotton plant height by mounting a 2D LiDAR and RTK-GPS on a high-clearance tractor to acquire the spatial coordinates of each point cloud (Ma et al., 2019). used a FastSCAN handheld scanner to obtain 3D point cloud data of maize and reconstructed the maize canopy. Phenotypic traits of the canopy were then calculated by fitting spheres and cylinders to the reconstructed structures.

With the rapid advancement of computing technologies, deep learning-based methods for processing 3D point cloud data have become a key approach for acquiring three-dimensional phenotypic information (Han et al., 2022). proposed a neighborhood spatial constraint method to filter out floating points and outlier noise from point clouds, and developed a new network called MIX-Net for point cloud segmentation. Compared with PointNet++ and DGCNN, MIX-Net achieved performance improvements of 3.1% and 1.7%, respectively (Guo et al., 2023). developed the ASAP-PointNet model, which was used for semantic segmentation of cabbage point clouds. The resulting phenotypic parameters—including plant height, leaf length, leaf width, and leaf area—showed correlation coefficients of 0.96, 0.91, 0.95, and 0.94 with the corresponding measured values (Shen et al., 2024). proposed a neural network-based algorithm for organ segmentation of cotton seedlings, achieving an average accuracy of 96.67%. The segmented leaf and stem point clouds were then used to calculate phenotypic parameters such as stem length, leaf length, leaf width, and leaf area.

In summary, traditional phenotypic measurement methods have numerous limitations and can no longer meet the demands of agricultural automation. As a result, 3D phenotypic acquisition based on deep learning neural network models has become a promising future direction, enabling rapid and accurate detection of the effects of salt stress on maize seedling growth. In this study, we propose an improved model named PointCornNet, based on PointNet++, which incorporates the CBAM dual-channel attention mechanism, replaces the original loss function with Varifocal Loss, and introduces the CronDBSCAN clustering algorithm to achieve both semantic and instance segmentation of point clouds while improving the accuracy of the original model. Phenotypic parameters of the segmented maize seedlings are calculated, and the strong linear relationships between the calculated results and the ground truth values are analyzed to validate the method’s efficiency and non-destructive capability in extracting key phenotypic traits. We further conducted a germination experiment under salt stress conditions and, based on the acquired seedling growth point clouds and the PointCornNet model, analyzed the variation patterns of different phenotypic traits of maize seedlings throughout their growth process under salt stress.

## 2 Materials and methods

### 2.1 Data collection

We selected plump, pest-free, and undamaged seeds of the Jinguan 597 maize variety for the germination experiment. The seed germination chamber used in the experiment consists of three main components: a seed cultivation module, an environmental control module, and a humane,mental interaction module. The seed cultivation module features a 3D-printed germination tray designed specifically for conducting maize germination trials. The environmental control module enables real-time regulation of temperature, humidity, and light within the chamber, with temperature adjustable between 10 °C and 50 °C and humidity ranging from 30% to 70%. The internal lighting system includes both growth and supplementary lamps. The human.mentary interaction module allows users to control environmental settings such as temperature, humidity, and lighting via a touchscreen interface mounted on the chamber.

**Figure 1:**
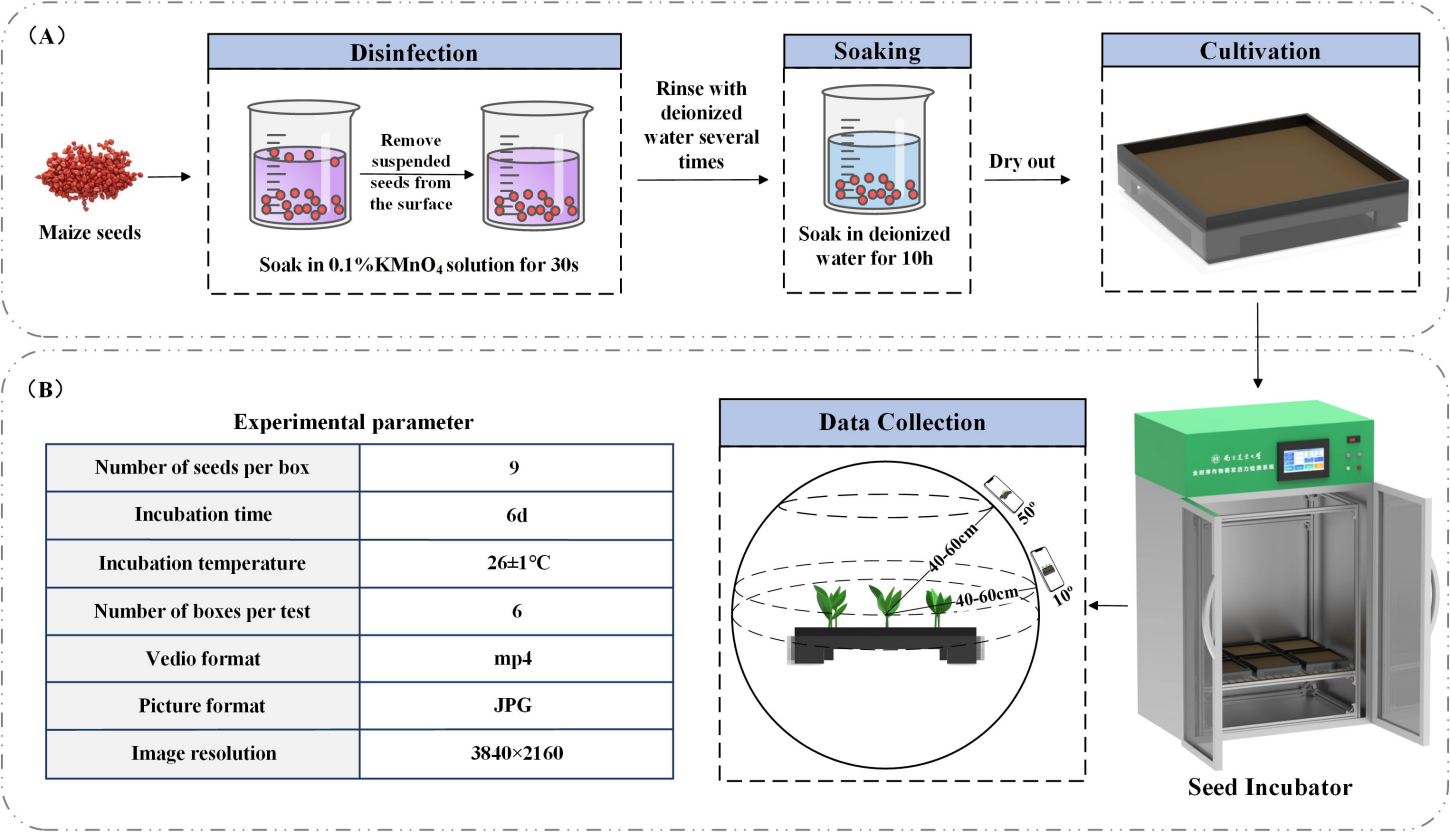
**(A)** Seed germination procedure; **(B)** experimental parameters.

The emergence of the first true leaf breaking through the soil by 5–10 mm was used as the indicator of the maize seedling stage. According to growth stages, maize typically enters the two-leaf and one-heart stage 5 to 7 days after emergence; thus, we selected day 6 after sowing as the experimental time point. Once the seedlings had emerged, we recorded videos of each plant using a smartphone camera every 12 hours. To minimize leaf occlusion, which could interfere with the 3D reconstruction, we selected the midpoint of the height of the plant located in the center of the tray as the focal point. The recording distance was maintained at 40–60 cm from this focal point, with plants recorded at two specific angles (10° and 50° relative to the ground). A complete 360° rotation was performed at each angle to ensure comprehensive coverage. Although manual operation introduced slight angular deviations within a 0-5° range, our verification confirmed these minor deviations did not significantly affect the 3D reconstruction quality. Each video lasted approximately two minutes and was later processed by extracting frames, yielding around 250 still images per video. The germination procedure is illustrated in **Figure 1A**, and the experimental parameters are shown in **Figure 1B**.

In order to verify the accuracy of the plant height and canopy width calculated by the model subsequently, manual measure was conducted after each recording is completed. For the plant height, a ruler was used to measure the distance from the above-ground part of the plant to the top of the plant canopy. For the canopy width, a caliper was used to measure the distance from the edge of the leaf on one side of the plant’s canopy to the opposite edge on the other side. When measuring each parameter, we repeated the measurement and took the average to obtain the measured value. In addition, the leaf was lightly touched during each measurement to prevent the distortion of the measured values due to the bending or displacement of the leaf.

### 2.2 Dataset construction

#### 2.2.1 3D Reconstruction

**Figure 2:**
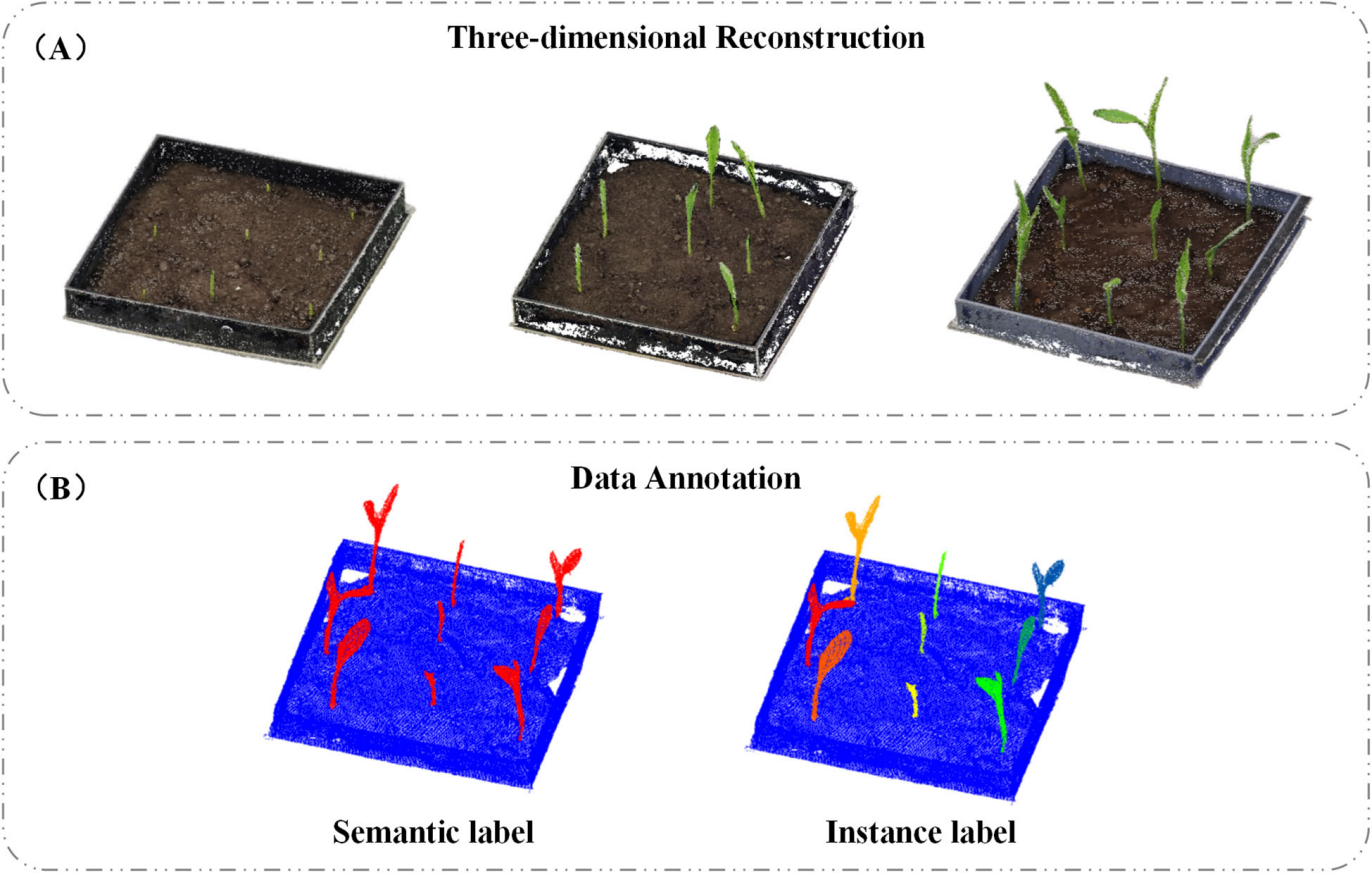
**(A)** 3D reconstruction; **(B)** data annotation.

Image-based 3D reconstruction is a technique that restores three-dimensional models from two-dimensional images (Aharchi and Ait Kbir, 2020). To obtain 3D point clouds of maize seedlings, we employed Agisoft Metashape (version 1.6, Agisoft LLC, St. Petersburg, Russia) to reconstruct 3D models from video-extracted image frames. This software primarily utilizes Structure-from-Motion (SfM) and Multi-View Stereo (MVS) algorithms to process image data. Specifically, the SfM algorithm applies Scale-Invariant Feature Transform (SIFT) (Lowe, 2004) to extract key points from the images and match them across multiple views. By establishing correspondences between these key points, SfM estimates the relative positions of the images and generates a sparse point cloud model while simultaneously solving for camera positions and intrinsic parameters (Chen et al., 2020). The MVS algorithm is then applied to densify the point cloud, using refined image alignment and reconstruction techniques to significantly enhance both the density and accuracy of the resulting point clouds (Aanæs et al., 2016). A total of 106 datasets were collected, resulting in 106 unique 3D point clouds. Selected examples of the reconstructed models are shown in **Figure 2A**.

#### 2.2.2 Data annotation

After obtaining the 3D point cloud data of maize seedlings, each dataset was meticulously annotated to facilitate subsequent model training. We used CloudCompare (Martinez-Guanter et al., 2019) to label the point clouds captured during different stages of maize seedling growth, as illustrated in **Figure 2B**. The annotated data were saved in.txt format, with each line representing a single point and containing the following information: x, y, z coordinates; Nx, Ny, Nz normals; and two labels—label1 and label2. Label1 was used for semantic segmentation, with values of 0 indicating non-seedling points and 1 indicating seedling points. Label2 was used to validate instance segmentation, where 0 denoted non-seedling points, and integers such as 1, 2, 3, etc., represented individual seedling instances.

#### 2.2.3 Data augmentation

To enhance the robustness and generalization capability of the network model, the annotated raw point clouds were augmented to increase both the quantity and diversity of training samples. The augmentation techniques applied in this study included: Fake Dropout, where 10% to 20% of points were randomly dropped; Jittering, which added Gaussian noise with a standard deviation of 0.01, constrained within ±0.05; Rotation, involving independent rotations along the X, Y, and Z axes within a range of [fngen 10nge Scaling, with random scaling factors between 0.67 and 1.5; Shuffling, achieved through index-based random reordering; and Translation, with random shifts applied in the range of [fngee 0.1] along each axis. These augmentation strategies effectively enriched the training dataset and improved the model’s ability to generalize across varied inputs. After augmentation, a total of 742 point clouds, including the original data, were generated. All.txt files were randomly split into training, testing, and validation sets in a ratio of 8:1:1, forming the dataset used for model training.

### 2.3 Point cloud segmentation model architecture based on PointNet++ (PointCornNet)

**Figure 3:**
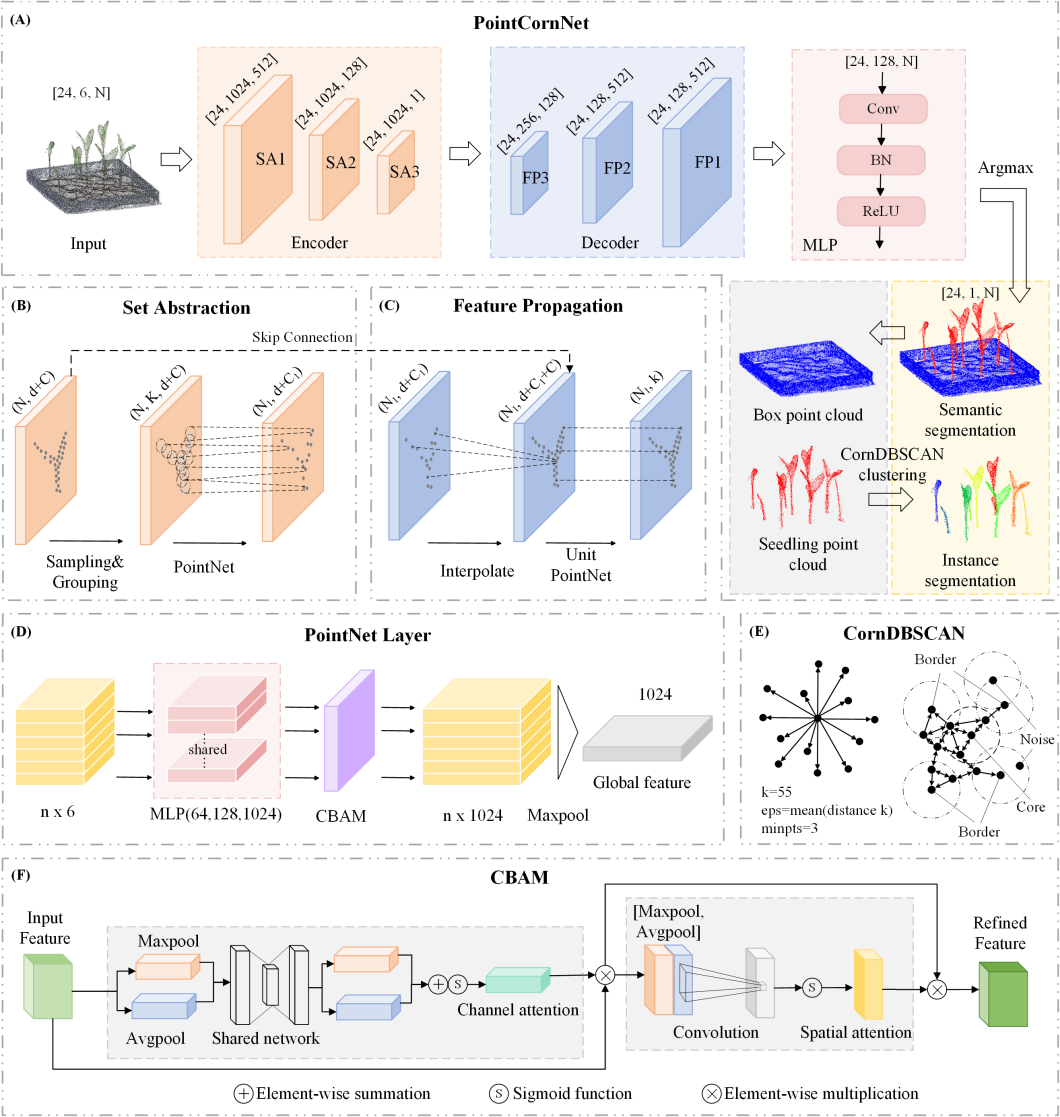
**(A)** Architecture of the PointCornNet network; **(B)** structure of the Set Abstraction (SA) module; **(C)** structure of the Feature Propagation (FP) module; **(D)** architecture of the PointNet layer; **(E)** CornDBSCAN clustering process; **(F)** CBAM attention module architecture.

The PointNet family is a crucial set of models in point cloud processing, leveraging spatial operations to handle the unordered and unstructured nature of point clouds for classification and segmentation tasks (Yuan et al., 2023). Compared to traditional deep learning models, such as voxel-based networks, PointNet offers several advantages. PointNet extracts features from each point using multilayer perceptrons (MLPs) and aggregates them through max pooling. PointNet++ is an enhanced version of PointNet that divides the point cloud into hierarchical layers, applying MLPs and max pooling at each layer to extract multi-level features, thereby capturing both global and local information in the point cloud. By combining features from multiple scales, it enhances robustness. The PointNet++_MSG variant, designed for semantic segmentation, uses Multi-Scale Grouping (MSG) to sample neighboring points at different scales, allowing the model to capture local geometric details more effectively. PointNet++_MSG has demonstrated particularly strong performance in semantic segmentation tasks, especially in complex and fine-grained scenarios (Qi et al., 2017). To better capture point cloud features and achieve both semantic and instance segmentation of maize seedlings, we propose an improved version of PointNet++_MSG, named PointCornNet. As shown in **Figure 3**, the input point cloud is processed through an encoder, decoder, and MLP layers to perform semantic segmentation. The segmented seedling point clouds are then subjected to a clustering algorithm for instance segmentation, resulting in the separation of individual seedlings. The specific improvements are outlined as follows:

(1)Integration of the CBAM Attention Mechanism (Woo et al., 2018). PointNet++ serves as the backbone network, consisting of an encoder and a decoder. The encoder comprises three cascaded Set Abstraction (SA) modules, with each SA module embedded with a corresponding CBAM module to capture salient features and spatial relationships within the point cloud data. Unlike the traditional CBAM used for 2D images, PointCornNet has made targeted adjustments to CBAM in response to the disorder, sparsity and 3D spatial distribution characteristics of point cloud data, enabling it to effectively capture the local geometric features and global dependencies of point clouds. Specifically, the original CBAM processes image features with a grid structure, whose dimensions are H×W×C (H and W are spatial dimensions, and C is the channel). In PointCornNet, CBAM processes point cloud features with dimensions of B×N×C (where B represents batch size, N represents the number of points and C represents feature channels). Each SA module includes Sampling, Grouping, and a PointNet Layer, where the CBAM mechanism is integrated within the PointNet Layer. As illustrated in **Figure 3D**, after the input of the PointNet Layer is processed through an MLP to generate a feature vector, this vector is passed into the CBAM module for a series of attention-based weighting operations. Subsequently, a local max pooling operation aggregates the focused local features into a global representation. As illustrated in **Figure 3F**, CBAM has two sequential sub-modules: channel attention and spatial attention. The overall process can be summarized as:


$$F_{1}=M_{c}(F)⊗F$$

$$F_{2}=M_{s}(F_{1})⊗F_{1}$$

Here, $F_{1}$ denotes the Channel weighted features. $M_{c}$ denotes the channel attention map. *F* denotes the input feature. ⊗ denotes element-wise multiplication. $M_{s}$ denotes the spatial attention map. $F_{2}$ denotes the refined feature.

In the channel attention module, average-pooling and max-pooling operations are first performed on the input feature, and then average-pooled features and max-pooled features are input into a shared network. After the shared network is applied to each pooled feature, we use element-wise summation and sigmoid function to compute the channel attention map. The channel attention map is computed as:


$$M_{c}(F)=\sigma (MLP(AvgPool(F))+MLP(MaxPool(F)))$$

Here, σ(·) denotes the sigmoid function. MLP(·) denotes the multilayer perceptron operation. AvgPool(·) denotes the average-pooling operation. MaxPool(·) denotes the max-pooling operation.

In the spatial attention module, we first apply average-pooling and max-pooling operations along the channel axis. After that, the pooled features are concatenated and convolved by a convolution layer. Finally, the spatial attention map is obtained through the sigmoid function. The spatial attention map is computed as:


$$M_{s}(F_{1})=\sigma (f([AvgPool(F_{1});MaxPool(F_{1})]))$$

Here, f(·) denotes the convolution operation.

CBAM comprises channel attention and spatial attention modules that adaptively weight features based on their relevance. Channel attention highlights the more critical channels in the point cloud segmentation task by learning the weight of each channel, and suppresses redundant and noisy channels such as irrelevant backgrounds. Spatial attention enhances the focus on key spatial positions such as the corners and edge points of seedlings by learning the weight of each point, while weakening irrelevant points. This mechanism enhances the network’s focus on critical information while suppressing less important or irrelevant features, thereby improving the model’s robustness against noise and extraneous data.

(2)Use of Varifocal Loss (Zhang et al., 2021). Given the characteristics of maize seedling point cloud data—specifically, the significant imbalance between seedling and non-seedling points, with non-seedling points greatly outnumbering seedling points—the original loss function used in the PointNet++ framework may overly favor the accurate prediction of non-seedling points. This imbalance can compromise the model’s overall performance and its ability to accurately identify seedling regions. To address this issue, we adopt Varifocal Loss, an improved version of the widely used Focal Loss (Lin et al., 2017). Varifocal Loss integrates both classification and regression components, dynamically adjusting weights to better handle class imbalance and localization inaccuracies. Specifically, it assigns higher weights to hard-to-classify samples, such as seedling points, thereby enhancing the model’s segmentation accuracy and localization precision for seedling regions. By incorporating Varifocal Loss, we not only achieve a better balance between the prediction of background and seedling points but also significantly improve model performance under imbalanced data distributions. This is particularly beneficial for enhancing recognition accuracy in seedling areas, ultimately optimizing the overall point cloud segmentation task. The Varifocal Loss is defined as follows:


$$L_{vf}=L_{cls}+\lambda \cdot L_{reg}$$

$$L_{cls}=-\frac{1}{N}\sum_{i=1}^{N}[y_{i}\log(p_{i})+(1-y_{i})\log(1-p_{i})]$$

$$L_{reg}=\sum_{i}|t_{i}-\bar{t_{i}}|$$

Here, *L_cls_* denotes the classification loss, and *L_reg_* represents the regression loss. *y_i_* is the ground truth label, and *p_i_* is the predicted class probability. *t_i_* and $\bar{t_{I}}$ refer to the ground truth bounding box and the predicted bounding box, respectively.

(3)Integration of CornDBSCAN for Instance Segmentation Clustering. In this study, to separate multiple maize seedling instances into individual plants, we adopt clustering as a simple yet effective method for instance segmentation. DBSCAN (Deng, 2020), a density-based clustering algorithm, is well-suited for this task due to its ability to detect clusters of arbitrary shapes and its strong robustness to noise. However, the performance of DBSCAN heavily depends on two key parameters: eps (the neighborhood radius) and minpts (the minimum number of points required to form a cluster). To enable adaptive clustering across varying datasets, we propose an optimized version of DBSCAN, termed CornDBSCAN, as illustrated in **Figure 3E**. The core idea involves using a Nearest Neighbors approach to compute the k-nearest neighbor distances for each point, where we set k = 55. For each point, the distance to its 55th nearest neighbor (i.e., the most distant neighbor within the k-nearest set) is selected as a density indicator. Subsequently, the mean of these 55th nearest neighbor distances across all points is computed and used as an estimated eps value. This process can be described as follows:


$$eps=\frac{1}{N}\sum_{i=1}^{N}distances[i,\;-1]$$

Here, distances refers to the computed k-nearest neighbor distance matrix, and distances[*i*, -1] denotes the distance from point i to its 55th nearest neighbor, i.e., the farthest neighbor within the top 55 nearest points.

Subsequently, the estimated eps value is applied to the DBSCAN clustering algorithm, with minpts set to 3, meaning that each cluster must contain at least three points. The point cloud data is then clustered using DBSCAN, and each point is assigned a cluster label accordingly. Noise points are automatically labeled as −1 by the algorithm. Finally, the Hungarian algorithm is used to perform optimal matching between the predicted labels and the ground truth labels, enabling the calculation of accuracy and other evaluation metrics.

### 2.4 Model training parameters and evaluation metrics

The experiments were conducted on a Windows 11 operating system, equipped with an Intel^®^ Xeon^®^ Gold 6248R @ 3.00GHz processor, 80 GB of RAM, and an NVIDIA GeForce RTX 4090 GPU with 24 GB of VRAM. The development environment was based on Python 3.8, with PyTorch 2.0 as the deep learning framework and CUDA version 11.8. The model training parameters are summarized in **Table 1**.

**Table 1:** Model parameter settings.

Parameter	Value
Epoch	100
Batch Size	24
Optimizer	Adam
Learning Rate	0.0005
Decay Rate	10−4
npoint	4096

To comprehensively evaluate the effectiveness of the model in segmenting maize seedling point clouds, we adopted several performance metrics. For semantic segmentation, we used Overall Accuracy (Acc), Precision (Pre), Recall (Rec), and Mean Intersection over Union (mIoU). For instance segmentation, the evaluation metrics included Precision (Pre), Recall (Rec), F1-score (F1), and Instance Intersection over Union (iiou).

Accuracy measures the overall performance of the model by reflecting the proportion of correctly predicted samples among all samples. Precision indicates the proportion of true positive predictions among all samples predicted as positive by the model. Recall represents the proportion of actual positive samples that are correctly predicted by the model. Mean Intersection over Union (mIoU) is a widely used metric in semantic segmentation tasks that evaluates the model’s segmentation performance across different classes. IoU measures the overlap between the predicted and ground truth regions, and mIoU is the average IoU across all classes. F1-score is the harmonic mean of Precision and Recall, providing a balanced measure between them. Instance Intersection over Union (iiou) assesses the degree of overlap between predicted and ground truth labels at the instance level. The specific calculation formulas are as follows:


$$Acc=\frac{Correct\;Predictions}{Total\;Predictions}=\frac{TP+TN}{TP+FP+TN+FN}$$

$$Pre=\frac{TP}{TP+FP}$$

$$Rec=\frac{TP}{TP+FN}$$

$$IoU_{C_{i}}=\frac{Intersection_{C_{i}}}{Union_{C_{i}}}=\frac{TP_{C_{i}}}{TP_{C_{i}}+FP_{C_{i}}+FN_{C_{i}}}$$

$mIoU=\frac{1}{N}\sum_{i=1}^{N}IoU_{C_{i}}$


$$F1=2\times \frac{Pre\times Rec}{Pre+Rec}$$

$$IoU_{I_{j}}=\frac{Intersection_{I_{j}}}{Union_{I_{j}}}=\frac{TP_{I_{j}}}{TP_{I_{j}}+FP_{I_{j}}+FN_{I_{j}}}$$

$$iiou=\frac{1}{M}\sum_{i=1}^{M}IoU_{I_{j}}\;$$

Here, True Positive (*TP*) refers to the number of samples correctly predicted as positive; False Positive (*FP*) refers to the number of samples incorrectly predicted as positive; True Negative (*TN*) refers to the number of samples correctly predicted as negative; and False Negative (*FN*) refers to the number of samples incorrectly predicted as negative. *N* denotes the total number of semantic classes, and ${IoU}_{C_{I}}$ represents the Intersection over Union for class *C_i_*. *M* denotes the total number of instances, and ${IoU}_{I_{j}}$ represents the Intersection over Union for instance *I_j_*.

### 2.5 Effectiveness of PointCornNet network on seedling datasets

In order to verify the feasibility of the proposed improved model PointCornNet in terms of performance, we compared the performance of the semantic segmentation module of the PointCornNet model with other semantic segmentation models, including DGCNN, PointNet, and PointNet++_MSG. We also compared the performance of the instance segmentation module of the PointCornNet model with other instance segmentation algorithms, including Euclidean Clustering, DFSP, and DBSCAN. When training different models and algorithms, all parameters remained consistent. The comparative test results of different semantic segmentation models are shown in **Table 2**, and the comparative test results of different instance segmentation algorithms are shown in **Table 3**.

**Table 2:** Comparative test results of semantic segmentation performances of network models.

Model	Acc/%	Pre/%	Rec/%	mIoU/%
DGCNN	93.76	91.38	75.92	89.13
PointNet	91.66	90.79	72.88	86.27
PointNet++_MSG	98.23	96.41	79.83	95.92
PointCornNet	99.74	97.45	87.62	97.23

**Table 3:** Comparative test results of instance segmentation performances of different algorithms.

Algorithm	Pre/%	Rec/%	F1/%	iiou/%
Euclidean Clustering	68.33	53.12	65.48	52.78
DFSP	64.61	60.18	60.45	59.07
DBSCAN	82.90	77.83	80.77	75.32
PointCornNet	97.44	95.00	94.93	92.24

**Figure 4:**
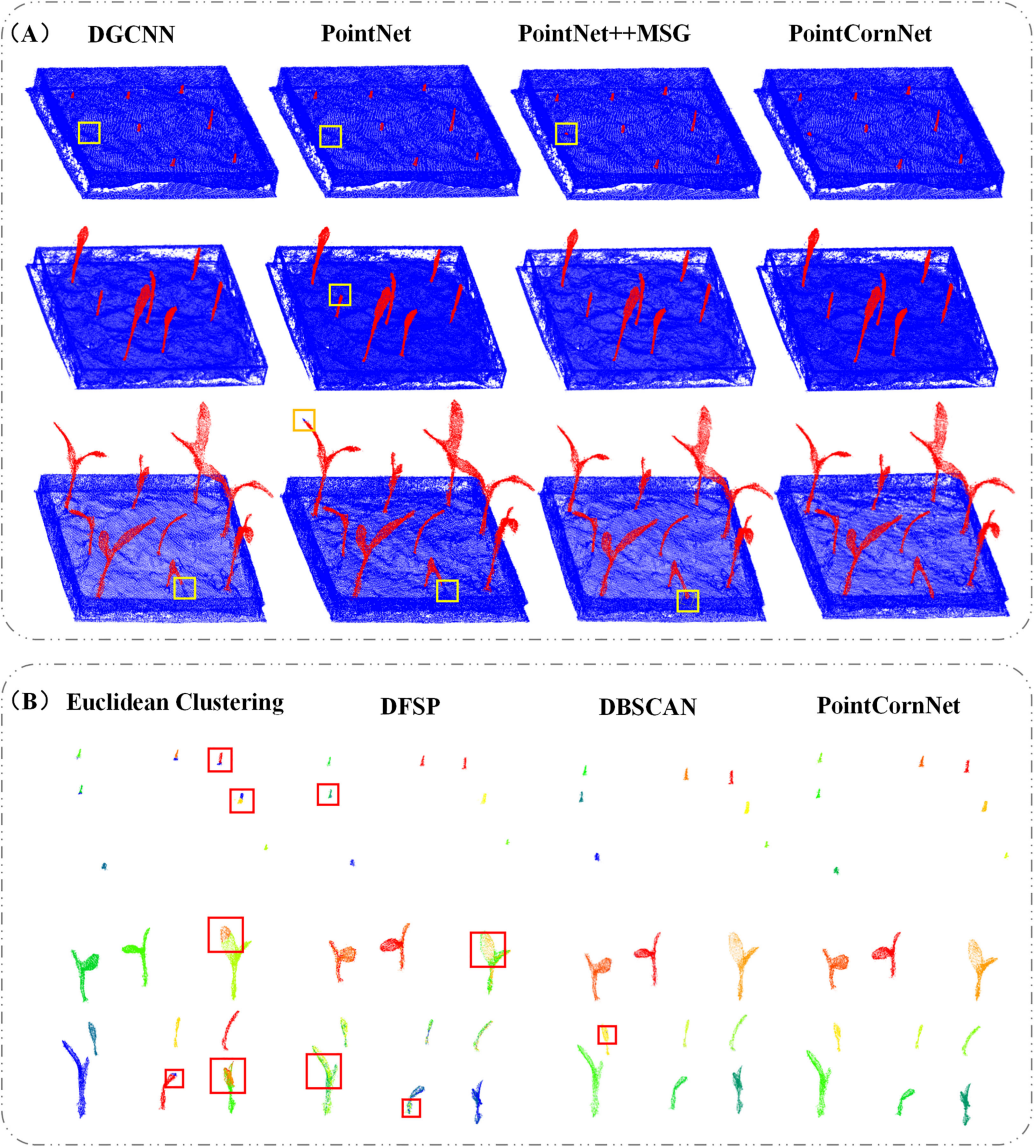
**(A)** Visualization of semantic segmentation results from different models; **(B)** visualization of instance segmentation results from different clustering algorithms.

The experimental results demonstrate that, compared with previous versions of PointNet, PointCornNet offers superior accuracy and stability. Additionally, the improved clustering algorithm CornDBSCAN achieves higher segmentation precision than other clustering methods, confirming that the PointCornNet model can effectively perform both semantic and instance segmentation on maize seedling point clouds. **Figure 4A** presents the segmentation visualizations of the four models at different growth stages of maize seedlings, where yellow boxes highlight areas of segmentation errors. **Figure 4B** shows the instance segmentation visualizations using the four clustering algorithms at various growth stages, with red boxes indicating segmentation errors.

### 2.6 Calculation of phenotypic parameters in corn seedlings

#### 2.6.1 Coordinate alignment and scale adjustment

Due to discrepancies between the reconstructed point cloud plane and the actual coordinate plane, various errors may arise, which can negatively impact the accuracy of phenotypic parameter calculations. To address this, we first standardized the z-axis orientation, aligning it to be perpendicular to the ground. This was achieved using the RANSAC algorithm to detect the ground plane and obtain its normal vector. The angle between this detected normal and the target z-axis was then calculated, and the Rodrigues’ rotation formula was used to derive the rotation matrix for point cloud alignment. To establish a correspondence between the plant point clouds in the 3D virtual space and the actual physical size of plants in the real world, a scaling factor was determined based on a known reference object. In this study, the seedling cultivation tray was used as a reference to compute the scale ratio. The specific calculation formula is as follows:


$$\theta =\cos^{-1}(\frac{m\cdot n}{\left|m|\times |n\right|})\;$$

$$R_{rot}=E\times \cos\theta +(m\cdot n)\times d\times (1-\cos\theta )+(m\cdot n)\times \sin\theta \;\;$$

$$k=\frac{L_{real}}{L_{virtual}}\;$$

Here, θ denotes the rotation angle, m is the normal vector of the actual ground plane, and n is the unit vector along the z-axis. *R_rot_* represents the rotation matrix, *E* is the 3×3 identity matrix, and d is the unit skew-symmetric matrix derived from the cross product of m·n . The scaling factor *k* is calculated based on the reference object, where *L_real_* is the real-world side length of the cultivation tray (25 cm), and *L_virtual_* ​ is the corresponding side length measured from the reconstructed model.

#### 2.6.2 Plant height calculation method

**Figure 5:**
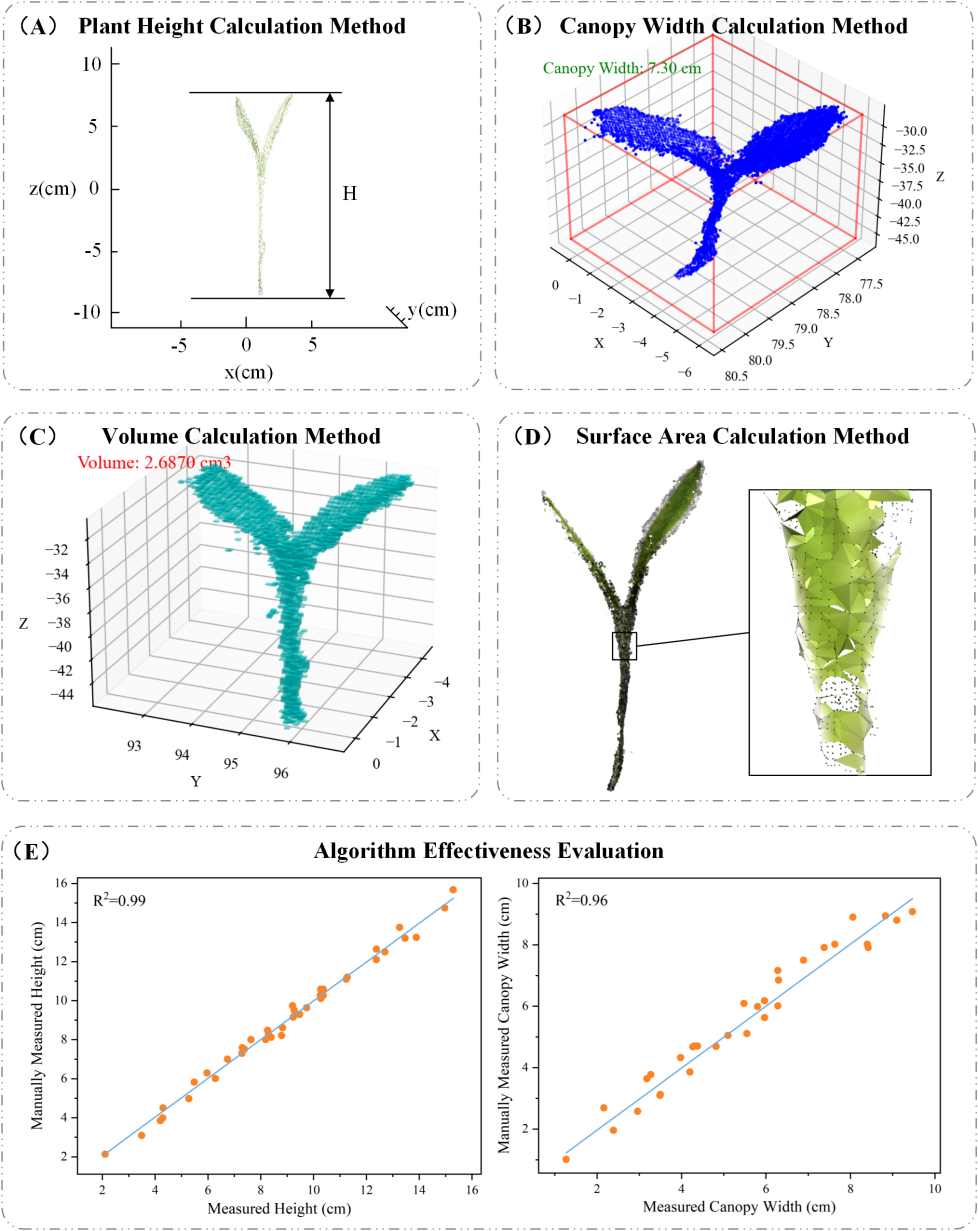
**(A)** Plant height calculation method; **(B)** canopy width calculation method; **(C)** volume calculation method; **(D)** surface area calculation method; **(E)** algorithm effectiveness evaluation.


Plant height is a key indicator for assessing plant growth, and its variation can be significantly influenced by environmental conditions (Tilly et al., 2014). In general, plant height is determined by measuring the vertical distance between the highest and lowest points of the plant. In this study, the height of maize seedlings is defined as the distance from the point where the plant contacts the soil to the top of the seedling. To compute this, all points within the maize seedling point cloud are traversed to identify the maximum and minimum values along the z-axis, and the height is calculated as the difference between these two values. A schematic illustration of the calculation is shown in **Figure 5A**. The formula is as follows:

$$\;H=z_{max}-z_{min}$$

Here, *H* represents the calculated seedling height, *z_max_* is the maximum value along the z-axis, and *z_min_* is the minimum value along the z-axis.

#### 2.6.3 Canopy width calculation method

Canopy width refers to the maximum horizontal span of a plant’s canopy—including leaves, stems, and other structures—on a horizontal plane. For maize seedlings, canopy width reflects the plant’s spatial expansion and serves as an important indicator of growth status, photosynthetic potential, and biomass accumulation (Guo et al., 2005). In this study, we constructed an Axis-Aligned Bounding Box (AABB) around the point cloud data and calculated the diagonal length of the bounding box using the Pythagorean theorem based on its length and width. This diagonal is used as an estimate of the canopy width, as illustrated in **Figure 5B**.

#### 2.6.4 Volume calculation method

As a key phenotypic parameter, 3D volume reflects not only the spatial occupancy of a plant but also correlates closely with its growth condition, health status, and biomass accumulation (Xiao et al., 2020). To accurately measure the volume of individual maize seedlings, we employed a voxel-based volume estimation method. First, an Axis-Aligned Bounding Box (AABB) was constructed around each point cloud, and the voxel size was determined based on the bounding box’s length, width, and height. The bounding box was then divided into a grid of small voxel units, and each point was assigned to its corresponding voxel. The total volume was calculated by counting the number of occupied voxels and multiplying this count by the volume of a single voxel. A visual representation of this process is shown in **Figure 5C**. The voxel-based volume calculation formula is as follows:


$$V_{a}=\sum_{i=1}^{n}V_{i}$$

Here, *V_a_* represents the voxel-based 3D volume of the seedling, *V_i_* is the volume of a single voxel, which was set to a cube with side length of 0.1 cm based on empirical testing, and n denotes the number of occupied voxels.

#### 2.6.5 Surface area calculation method

Surface area is closely related to various physiological processes, crop health, photosynthetic efficiency, and resource utilization. In this study, we estimated the surface area of maize seedlings using the Ball Pivoting Algorithm (BPA) based on point cloud data. BPA is a surface reconstruction technique that generates triangular meshes from point clouds by simulating the motion of a virtual ball “rolling” across the data points. As the ball rolls over the surface, it identifies three-point contacts to form triangles, gradually constructing a surface mesh. Once the mesh is generated, the total surface area is calculated by summing the areas of all the triangular facets. A visual example of this process is shown in **Figure 5D**. The detailed calculation procedure is as follows:


$$M=\cup_{i=1}^{N}T(p_{i},r)$$

$$A=\sum_{j=1}^{k}S_{j}$$

Here, *M* denotes the generated mesh, *p_i_* is a point in the point cloud, and *r* is the radius of the rolling ball, which was empirically set to 0.2 cm. *T*(*p_i_*, *r*) represents the local triangular mesh formed by a ball of radius r centered at point *p_i_*. The total surface area *A* is computed by summing the areas of all individual triangles, where *k* is the total number of triangles and *S_j_* is the area of the *j* triangle. The area of each triangle can be calculated using Heron’s formula.

#### 2.6.6 Algorithm effectiveness evaluation

In this study, to evaluate the effectiveness and practicality of the proposed algorithm, we selected plant height and canopy width from 40 maize seedlings as assessment parameters. **Figure 5E** illustrates the relationship between the values obtained using the PointCornNet model and those obtained through manual measurements. The x-axis represents manually measured values, while the y-axis corresponds to the algorithm-derived measurements. As shown in the figure, the data points are predominantly distributed along the diagonal, indicating a strong agreement between the two sets of measurements. The coefficient of determination (R^2^) for plant height and canopy width reached 0.99 and 0.96, respectively, demonstrating a high correlation between algorithm-predicted and manually measured values. It is necessary to clarify that the calculation results of volume and surface area in this study were mainly used to analyze the dynamic changes of maize seedlings under salt stress. Their accuracy was not verified through manual measurement values. The interpretation of the relevant results should be limited to the specific analysis scope of this study.

## 3 Results

### 3.1 Experimental design

Using the improved point cloud segmentation model PointCornNet and the phenotypic trait calculation algorithm, we analyzed the dynamic changes in plant height, canopy width, volume, and surface area of maize seedlings under salt stress during the first six days after sowing. Salt stress conditions were simulated using NaCl solutions at six concentration levels: 0, 60 mmol·L^-1^, 120 mmol·L^-1^, 180 mmol·L^-1^, 240 mmol·L^-1^, and 300 mmol·L^-1^. For each concentration, plant 9 seeds in each experiment. The experiment was repeated three times, with 27 plants at each concentration, and the total sample size was 162 plants (Turan et al., 2009; He et al., 2024). Since no seedlings emerged within the first 36 hours after sowing, the first data collection was conducted at 48 hours post-sowing. Subsequent data were collected every 12 hours, with the final collection taking place at 144 hours after sowing.

### 3.2 Effects of different salt stress concentrations on the phenotypic traits of maize seedlings

Salt stress is one of the major factors affecting crop growth and yield, as saline soils can disrupt plant physiological and metabolic processes, thereby impairing seed germination (Shahid et al., 2020). However, the dynamic changes in multiple phenotypic traits of maize seedlings under salt stress conditions remain poorly understood. In this study, we systematically measured and compared plant height, canopy width, volume, and surface area of maize seedlings grown under six NaCl solution concentrations: 0, 60mmol·L^-1^, 120 mmol·L^-1^, 180 mmol·L^-1^, 240 mmol·L^-1^, and 300 mmol·L^-1^. This analysis aimed to investigate the impact of salt stress on maize seedling growth and provide experimental evidence for breeding salt-tolerant cultivars.

**Figure 6:**
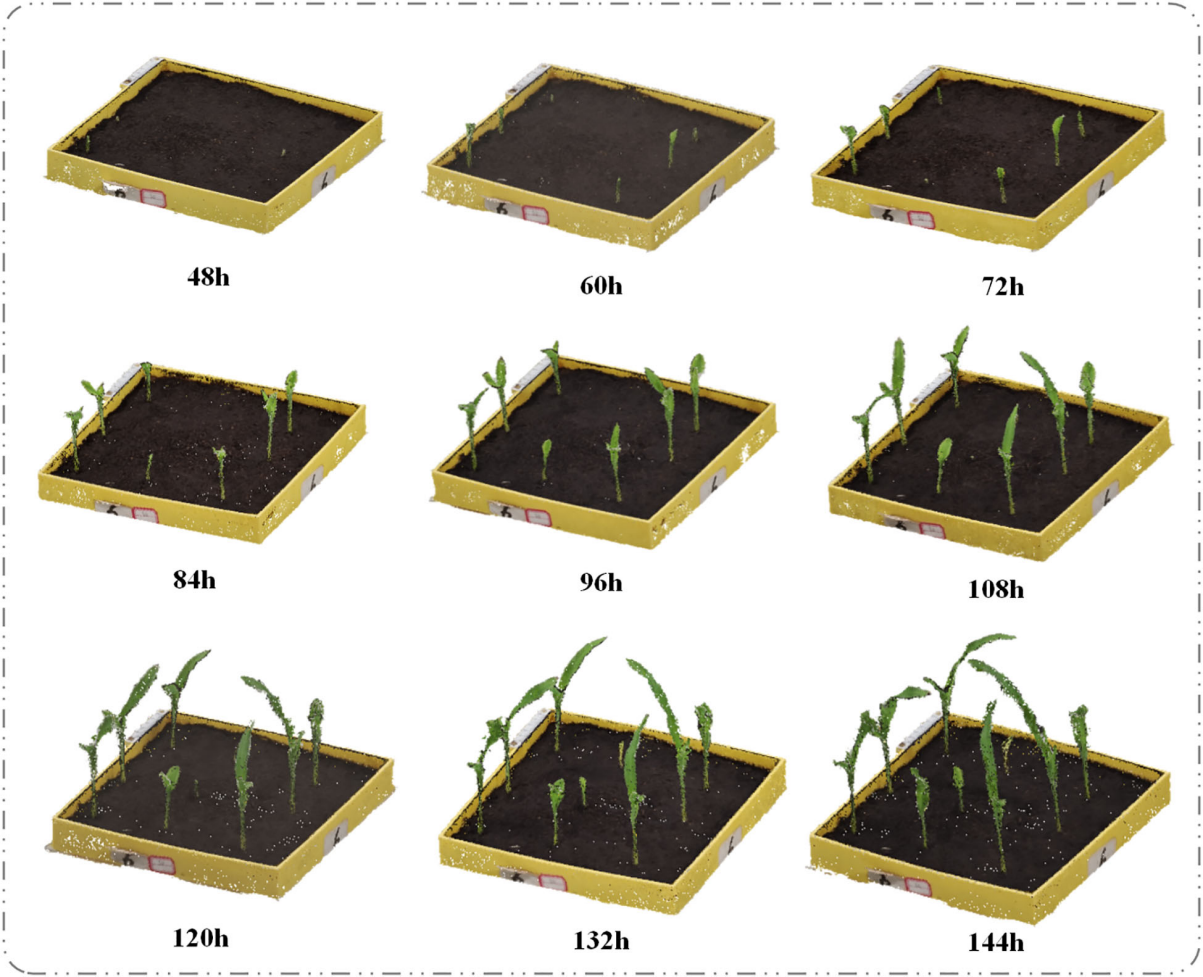
Temporal changes in the 3D point cloud of maize seedlings under CK (control) treatment.

**Figure 7:**
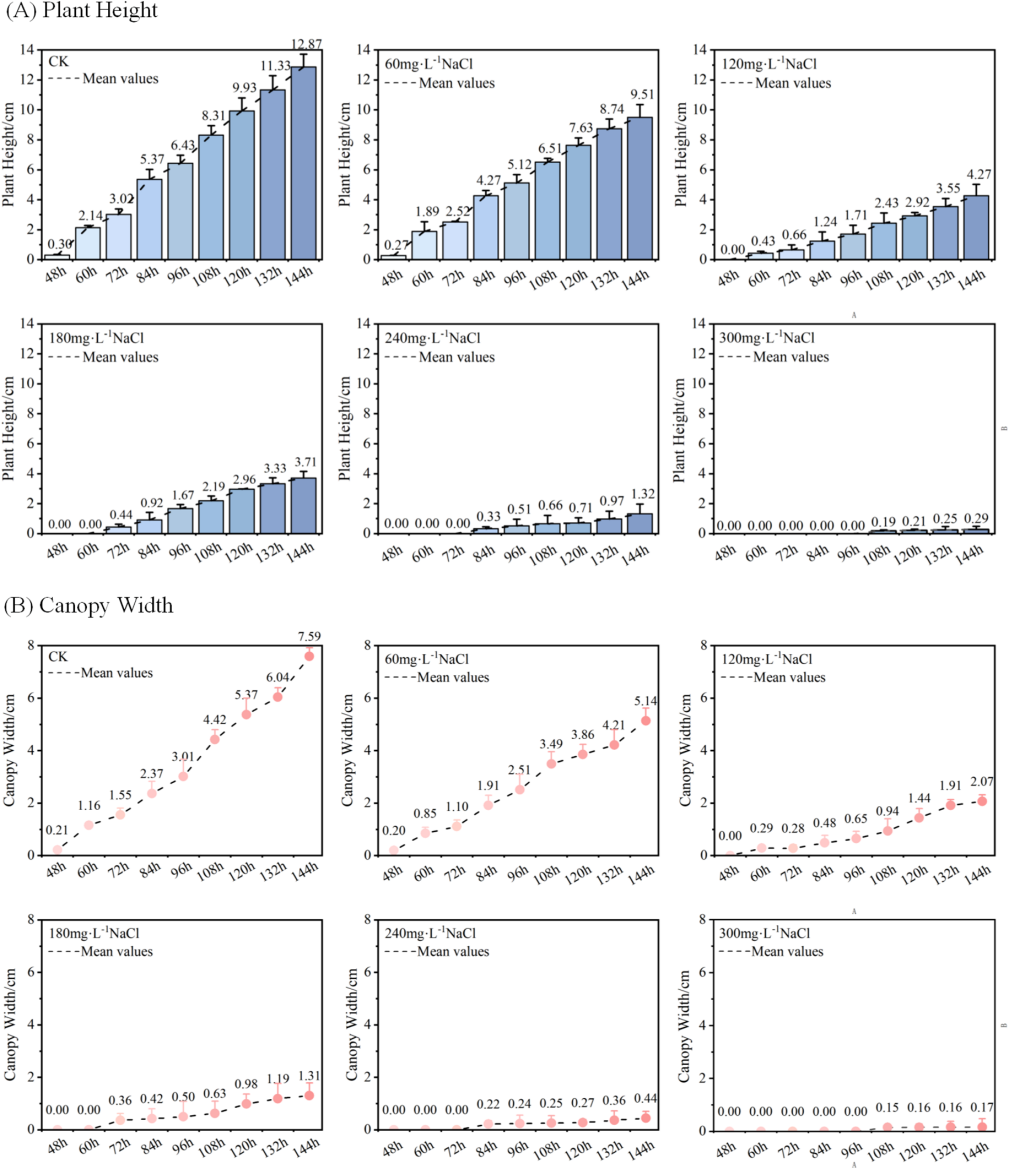
**(A)** Temporal changes in average plant height of maize seedlings under different NaCl concentration treatments; **(B)** Temporal changes in average canopy width of maize seedlings under different NaCl concentration treatments.

(1) Effects of Different Salt Stress Concentrations on Plant Height and Canopy Width of Maize Seedlings. We conducted maize seed germination experiments under CK (control), 60mmol·L^-1^, 120 mmol·L^-1^, 180 mmol·L^-1^, 240 mmol·L^-1^, and 300 mmol·L^-1^ NaCl treatments, and obtained 3D models of the seedlings during the first six days after sowing. The temporal 3D reconstruction of maize seedlings under CK treatment from 48h to 144h post-sowing is shown in **Figure 6**. Using the PointCornNet model and the phenotypic parameter computation algorithm, we measured the 3D models throughout the germination process and obtained the average plant height and canopy width of maize seedlings over time under different NaCl concentrations, as illustrated in **Figure 7**.

**Figure 7A** shows the temporal variation in average plant height of maize seedlings cultivated under different NaCl concentrations. Taking the data at D_t_ = 48h as an example, the “Mean Value” line represents the average of three replicate measurements of seedling height at 48 hours after sowing. As illustrated in **Figure 7A**, the average plant height increased over time across all treatment groups. However, as the concentration of NaCl increased, both the average plant height and its growth rate exhibited a declining trend. In addition, the initial emergence time of seedlings was progressively delayed under higher salt concentrations. At D_t_ = 144h, the average plant heights under different treatments were as follows: 12.87 cm (control, deionized water), 9.51 cm (60mmol·L^-1^), 4.27 cm (120mmol·L^-1^), 3.71 cm (180mmol·L^-1^), 1.32 cm (240mmol·L^-1^), and 0.29 cm (300mmol·L^-1^). These results clearly indicate that increasing NaCl concentration gradually inhibits the vertical growth of maize seedlings.

**Figure 7B** illustrates the changes in average canopy width of maize seedlings over time under different NaCl concentration treatments. Taking the data at D_t_ = 48h as an example, the “Mean Value” line represents the mean of average canopy widths across three replicate groups measured at 48 hours after sowing. As shown in **Figure 7B**, the canopy width of maize seedlings increased progressively with cultivation time in all treatment groups. However, as the NaCl concentration increased, both the overall canopy width and its growth rate exhibited a decreasing trend. At D_t_ = 144h, the average canopy widths of maize seedlings under the six treatments were as follows: 7.59 cm (control, deionized water), 5.14 cm (60mmol·L^-1^), 2.07 cm (120mmol·L^-1^), 1.31 cm (180mmol·L^-1^), 0.44 cm (240mmol·L^-1^), and 0.17 cm (300mmol·L^-1^). These results clearly demonstrate that increasing NaCl concentration gradually suppresses the lateral growth of maize seedlings, as reflected by reduced canopy width.

**Figure 8:**
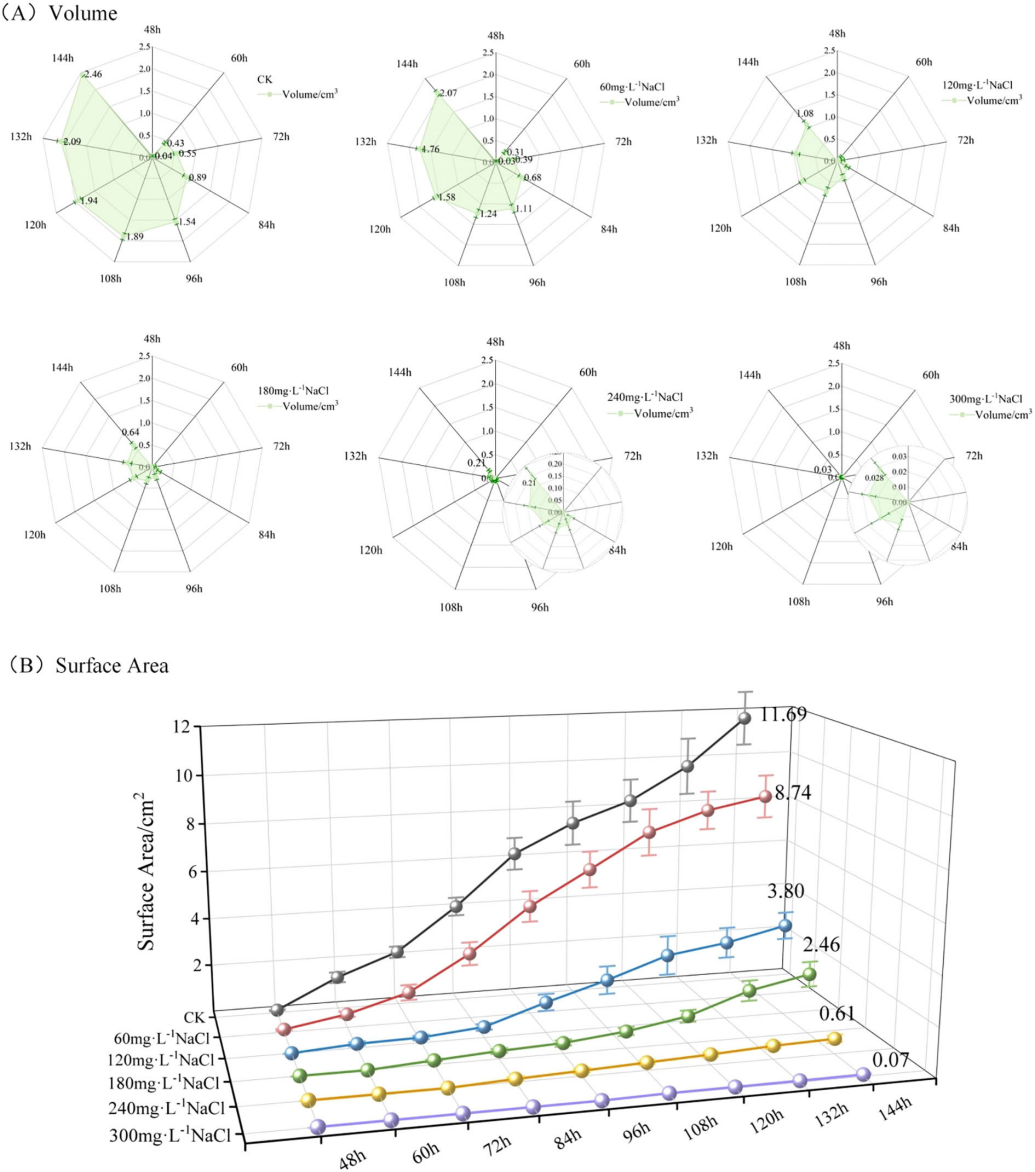
**(A)** Temporal changes in average volume of maize seedlings under different NaCl concentration treatments; **(B)** temporal changes in average surface area of maize seedlings under different NaCl concentration treatments.

(2) Effects of Different Salt Stress Concentrations on the Volume and Surface Area of Maize Seedlings. Using the PointCornNet model and the phenotypic trait computation algorithm, we analyzed the 3D models of maize seedlings throughout the germination process. The average volume and surface area of seedlings under different NaCl concentration treatments over time were obtained, as shown in **Figure 8**.

**Figure 8A** depicts the changes in maize seedling volume over time under different NaCl concentration treatments. Taking D_t_ = 48h as an example, the shaded area in the radar chart represents the average volume across three replicate groups measured at 48 hours after sowing. As illustrated in **Figure 8A**, the volume of maize seedlings increased over time under all treatment conditions. However, with increasing NaCl concentration, both the overall volume and its growth rate exhibited a downward trend. At D_t_ = 144h, the average volumes of maize seedlings under each treatment were as follows: 2.46 cm³ (control, deionized water), 2.07 cm³ (60mmol·L^-1^), 1.08 cm³ (120mmol·L^-1^), 0.64 cm³ (180mmol·L^-1^), 0.21 cm³ (240mmol·L^-1^), and 0.03 cm³ (300mmol·L^-1^). These results clearly demonstrate that increasing NaCl concentration progressively inhibits the volumetric growth of maize seedlings.

**Figure 8B** illustrates the changes in surface area of maize seedlings over time under different NaCl concentration treatments. Taking D_t_ = 48h as an example, the 3D line plots represent the mean surface area across three replicate groups measured 12 hours after emergence. As shown in **Figure 8B**, the surface area of maize seedlings increased steadily over time under all treatment conditions. However, with increasing NaCl concentration, both the overall surface area and its growth rate showed a clear decreasing trend. At D_t_ = 144h, the average surface areas of maize seedlings under each treatment were as follows: 11.69 cm² (control, deionized water), 8.74 cm² (60mmol·L^-1^), 3.80 cm² (120mmol·L^-1^), 2.46 cm² (180mmol·L^-1^), 0.61 cm² (240mmol·L^-1^), and 0.07 cm² (300mmol·L^-1^). These results clearly indicate that higher NaCl concentrations progressively suppress the surface area expansion of maize seedlings.

In summary, the proposed method for analyzing the full temporal sequence of maize seedling phenotypic changes based on the PointCornNet point cloud segmentation model is expected to provide valuable insights into the growth process of crop seeds under salt stress. This approach offers a deeper understanding of how salt stress influences crop phenotypic traits and internal physiological characteristics, and supports the development of salt-tolerant crop varieties. Ultimately, it contributes to the advancement of digital, modern agriculture and the pursuit of high-quality, high-yield crop production.

## 4 Discussion

To address the limitations of traditional manual measurements—namely low efficiency, high error rates, and potential damage to seedlings—and to meet the need for non-destructive monitoring throughout the entire growth process of maize seedlings, the following work was carried out:

1. Maize seedlings were cultivated using a seed incubator, and their growth process was recorded with a smartphone camera. Image frames were extracted from the recorded videos and used to perform 3D reconstruction, resulting in the construction of a labeled point cloud dataset for maize seedlings. In parallel, maize seedling cultivation experiments were conducted under salt stress conditions, and full-sequence 3D point cloud data were collected from 48 to 144 hours after sowing.
2. The PointNet++ point cloud segmentation model was improved and adapted for maize seedling segmentation, resulting in the proposed PointCornNet model. The key enhancements include the integration of the CBAM attention mechanism, the replacement of the original loss function with Varifocal Loss, and the incorporation of the CronDBSCAN clustering algorithm after semantic segmentation. The performance of the improved PointCornNet model was then evaluated through comparative experiments against other semantic segmentation models, including DGCNN, PointNet, and PointNet++_MSG, as well as against instance segmentation algorithms such as Euclidean Clustering, DFSP, and DBSCAN, validating the effectiveness of the proposed improvements.
3. Four phenotypic traits—plant height, canopy width, volume, and surface area—were selected as key measurement indicators, and specific computational methods were developed for each. The PointCornNet model was used to perform phenotypic parameter extraction on the segmented maize seedlings. Results showed that the calculated plant height and canopy width exhibited strong correlations with the manually measured values, with coefficients of determination (R^2^) of 0.99 and 0.96, respectively. These findings confirm that the proposed method can efficiently and non-destructively extract critical phenotypic traits.
4. Based on the PointCornNet model and the phenotypic trait calculation algorithm, the 3D models of maize seedlings during the growth process were analyzed. The study examined the temporal changes in plant height, canopy width, volume, and surface area within six days after sowing under different NaCl concentrations: control (CK), 60 mg·L^-1^, 120 mg·L^-1^, 180 mg·L^-1^, 240 mg·L^-1^, and 300 mg·L^-1^. The results revealed that as the NaCl concentration increased, all four phenotypic indicators and their growth rates showed a declining trend. In addition, the initial emergence time of seedlings was progressively delayed. These findings demonstrate that salt stress significantly inhibits the growth of maize seedlings, and the inhibitory effect intensifies with increasing NaCl concentration.

In summary, the non-destructive phenotypic detection method for maize seedlings based on the PointCornNet point cloud segmentation model provides effective support for phenotypic analysis throughout the seedling growth process. It also offers a powerful tool for the quantitative study of plant growth inhibition under salt stress. Furthermore, this method holds significant potential as a reference for the breeding of salt-tolerant crops, thereby contributing to the advancement of smart agriculture and crop breeding technologies.

## Data Availability

The raw data supporting the conclusions of this article will be made available by the authors, without undue reservation.
